# Statutory Premarital Haemoglobinopathy Screening, Marriage Decisions, and Affected Births: A Systematic Review

**DOI:** 10.7759/cureus.116974

**Published:** 2026-09-26

**Authors:** Fatimah Y Qumairy, Ghufran A Almowalad, Walaa H Albalawi, Jumanh F Al-hadeir, Remaz R Remely, Luna B Thaniane, Saleh A Alimam, Hassan M Al Abdulmohsen, Abdulmalik K Albalawi, Ragad S Alatawi, Abdullah A Al-Hussain

**Affiliations:** 1 Pediatrics, Jazan General Hospital, Jazan, SAU; 2 Faculty of Medicine, Jazan University, Jazan, SAU; 3 Faculty of Medicine, University of Tabuk, Tabuk, SAU; 4 College of Medicine and Health Sciences, Arabian Gulf University, Manama, BHR; 5 Faculty of Medicine, Qassim University, Qassim, SAU; 6 College of Medicine, King Faisal University, Al-Ahsa, SAU

**Keywords:** affected births, genetic counselling, haemoglobinopathies, marriage decisions, premarital screening, prenatal diagnosis, public health, sickle cell disease, termination of pregnancy, thalassaemia

## Abstract

Statutory premarital screening for haemoglobinopathies has been implemented in several Middle Eastern countries and elsewhere to identify couples at reproductive risk, but its effectiveness in reducing affected births remains uncertain. We conducted a systematic review and meta-analysis of studies evaluating statutory premarital screening for any haemoglobinopathy, searching MEDLINE, Cochrane Central Register of Controlled Trials (CENTRAL), Scopus, and Web of Science from 2000 onward. The primary outcome was the proportion of at-risk couples who did not proceed with marriage; the secondary outcome was change in affected births following programme implementation. Of 7,479 records screened after deduplication, 11 studies evaluating 7 programmes in 7 countries or territories were included. Ten studies contributed to the primary outcome, representing 14,906 at-risk couples. The proportion of couples who did not proceed with marriage varied substantially, with a pooled estimate of 33.6% (95% confidence interval, 16.3-56.6%; I²=99.1%). Three programmes reported reductions of 75%-100% in affected births following implementation, although these uncontrolled comparisons had important methodological limitations. Overall certainty of evidence was very low. Statutory premarital haemoglobinopathy screening is associated with substantial variation in subsequent marriage decisions, while affected births declined in programmes reporting longitudinal data. However, marked heterogeneity and risk of bias preclude reliable estimates of programme effectiveness or causal attribution of reductions in affected births to screening.

## Introduction and background

Inherited haemoglobin disorders are among the most common severe monogenic diseases worldwide and represent a substantial global health burden. Sickle cell disease and thalassaemia account for a large proportion of affected births and mortality [1,2]. Because these disorders are predominantly autosomal recessive, couples in whom both partners carry a pathogenic variant are at risk of having an affected child. Carrier frequencies are particularly high in the Arab world and the wider Eastern Mediterranean, where consanguineous marriage is also common, making identification of at-risk couples an important and potentially tractable prevention strategy [1-4].

Carrier screening can be undertaken before marriage, before conception, during pregnancy, or at birth. Premarital screening is distinctive because it identifies genetic risk before a couple marries and, in many jurisdictions, has been incorporated into statutory or quasi-statutory programmes. Such programmes have been implemented across the Middle East and neighbouring regions [5-9]. However, in most programmes, the legal requirement is to undergo testing rather than to avoid marriage following an incompatible result. Thus, premarital screening may prevent affected births through two different pathways: couples may decide not to marry after learning their reproductive risk, or couples may marry but use subsequent reproductive options, including prenatal diagnosis and termination, preimplantation genetic testing, or other reproductive choices [3,5,7]. These pathways are not interchangeable because their availability depends on the legal, religious, cultural, and healthcare context. Existing reports suggest substantial variation in marriage decisions following an at-risk result, but these findings have not been systematically synthesised [10,11].

Previous reviews have focused on specific haemoglobinopathies, regions, screening uptake, knowledge and attitudes, or genetic risk assessment and have not quantitatively synthesised either the proportion of at-risk couples who do not proceed with marriage or changes in affected births following programme introduction [1,4,6]. Evidence on the effectiveness and mechanisms of statutory premarital screening therefore remains uncertain. We undertook a systematic review and meta-analysis across all haemoglobinopathies and regions to estimate the proportion of at-risk couples identified by premarital screening who do not proceed with marriage and to characterise changes in the incidence of affected births before and after programme introduction. We also sought to determine which of the two potential pathways -- altered marriage decisions or reproductive interventions after marriage -- accounts for observed reductions in affected births.

## Review

Methods

Eligibility Criteria

The review was conducted according to the Preferred Reporting Items for Systematic Reviews and Meta-Analyses (PRISMA) 2020 statement [12]. We included studies of individuals or couples undergoing statutory premarital screening for haemoglobinopathies, defined as screening required by law or an established religious authority before marriage. National, regional, and governorate programmes were eligible; voluntary and non-statutory programmes were excluded.

All haemoglobinopathies and screening methods were eligible, with or without genetic counselling. The primary outcome was the proportion of at-risk couples who did not proceed with marriage, defined as couples in which both partners were carriers. The secondary outcome was the incidence or number of affected births reported over time or before and after programme implementation. Other programme outcomes were also extracted when available.

Eligible designs included programme cohorts, before-and-after studies, interrupted time series, cross-sectional reports, comparative studies, registry analyses, and randomised trials. Publications from 2000 onward were eligible without language or publication status restrictions. Studies were excluded if they involved non-statutory or non-premarital screening, did not report a review outcome, duplicated a more complete report, or were otherwise ineligible.

Information Sources and Search Strategy

MEDLINE via PubMed, Cochrane CENTRAL, Scopus, and Web of Science Core Collection were searched from inception, without language or publication status restrictions. The search combined terms for premarital screening, marriage, haemoglobinopathies, carrier status, and programme outcomes. Two complementary search strategies were used to capture studies without explicit premarital terminology. The strategy was validated against known programme evaluations.

Study Selection and Data Extraction

After deduplication, two reviewers independently screened titles, abstracts, and full texts, resolving disagreements through discussion or third-reviewer arbitration. Non-English records were translated when necessary.

Two reviewers independently extracted study, programme, population, screening, marriage, and affected birth data using a piloted form. Denominator ambiguities, outcome definitions, overlapping cohorts, and data provenance were recorded. No outcome values were inferred or reconstructed.

Overlapping Reports

For overlapping reports from the same programme, the report with the most complete data was used as the index report. Participants were not counted more than once for a given estimate.

Risk of Bias

Two reviewers independently assessed risk of bias. The Joanna Briggs Institute checklist for prevalence studies was used for the primary outcome and Risk of Bias in Non-randomised Studies of Interventions (ROBINS-I) for the secondary outcome [13,14]. Potential confounding of affected birth outcomes by changes in prenatal diagnosis, termination, screening, treatment, and consanguinity was considered.

Results

Study Selection

The final selection process is presented in Figure 1. Eleven studies were included, representing seven statutory premarital screening programmes across seven countries or territories. Ten studies contributed to the primary outcome and three to the secondary outcome, with two contributing to both. Three programmes were represented by multiple reports: the Kurdistan Region programme by three governorate-level reports, the Saudi programme by national and Jazan Province reports, and the Iranian programme by reports from Fars and Mazandaran. These reports involved disjoint populations and were therefore not double-counted, although they were not considered independent replications. A substantial proportion of reports were adjudicated at the abstract level because full texts were unavailable or no eligible outcome was identified. Several values initially extracted from abstracts were subsequently corrected following full-text review.

**Figure 1:**
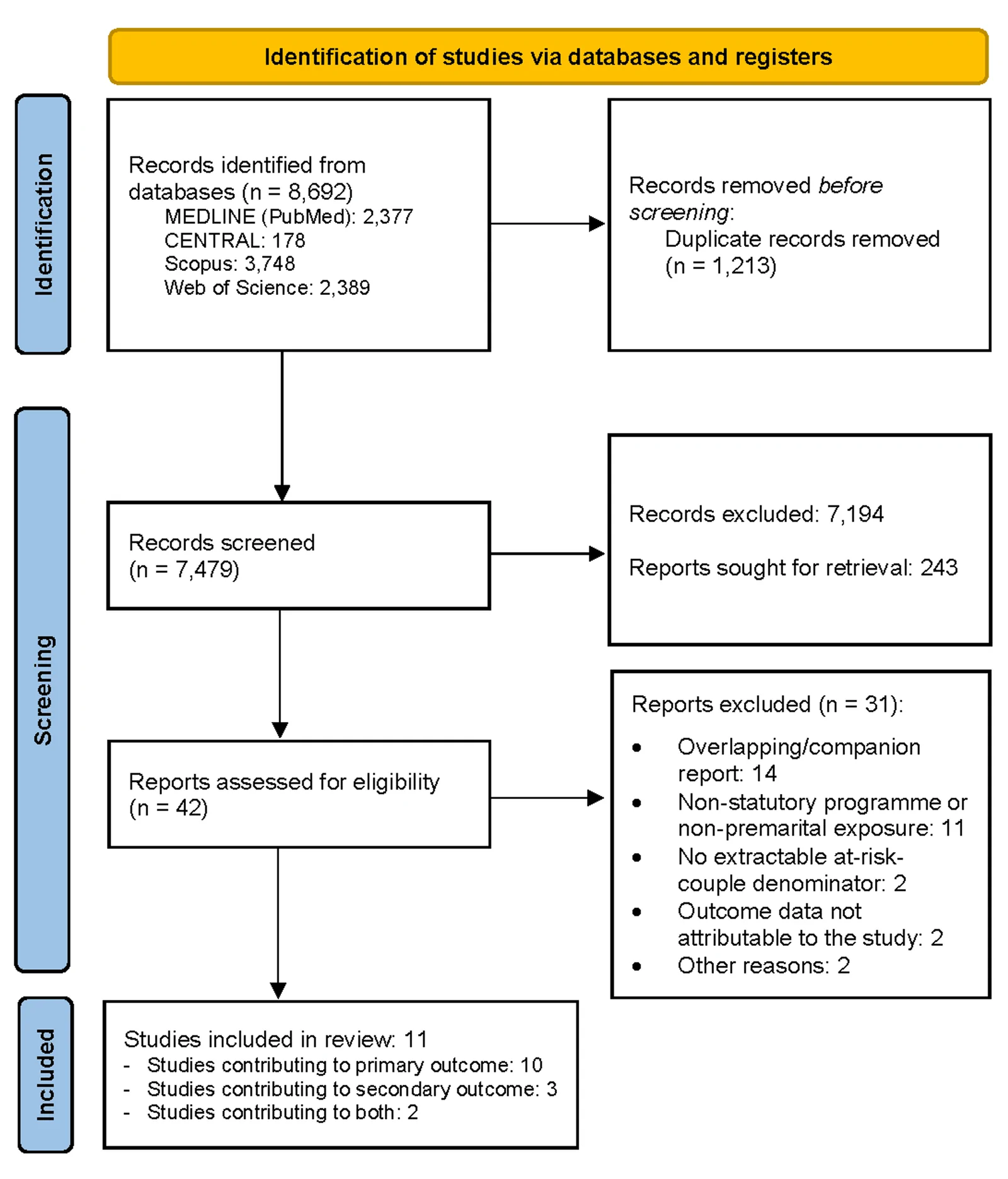
PRISMA flow diagram depicting the study selection process for the systematic review Following the identification of records through database searching and other sources, duplicates were removed, and the remaining records were screened. Full-text articles were assessed for eligibility, with exclusions documented along with reasons. Studies meeting the inclusion criteria were included in the final synthesis. PRISMA, Preferred Reporting Items for Systematic Reviews and Meta-Analyses.

Characteristics of Included Studies

Included studies described statutory premarital screening programmes in Bahrain, Northern Cyprus, Iran, Iraq, Kuwait, Palestine (Gaza), and Saudi Arabia. The statutory instrument was a national law or royal decree in most programmes, regional legislation in one, and a binding judicial decision in Gaza. Publication years spanned 2007 to 2025, and data collection spanned 1993 to 2023 (Table 1).

**Table 1:** Characteristics of included studies evaluating statutory premarital haemoglobinopathy screening programmes Characteristics of the 11 included studies evaluating statutory premarital screening programmes for haemoglobinopathies. The table summarises the study, country or region, study design, study period, haemoglobinopathy conditions assessed, statutory basis of the screening programme, number of at-risk couples, and outcomes evaluated. An at-risk couple was defined as a couple in which both partners were carriers and therefore had a 25% risk of having an affected child in each pregnancy. “Both” indicates that the study evaluated both the primary outcome (at-risk couples who did not proceed with marriage) and the secondary outcome (incidence of affected births before versus after programme implementation). NR, not reported.

Study	Country or region	Design	Period	Conditions	Statutory basis	At-risk couples	Outcome
Bozkurt (2007) [1]	Cyprus (North)	Programme report	Pre-programme vs since 2002	α- and β-thalassaemia	Compulsory by law, North Cyprus, 1980	—	Secondary
Tarazi et al. (2007) [2]	Palestine (Gaza)	Programme cohort with birth registry	September 2000-2005	β-thalassaemia	Decision of the superior legitimate judge, 9 September 2000	122	Both
Memish and Saeedi (2011) [3]	Saudi Arabia	Programme cohort	2004-2009	β-thalassaemia and sickle cell	Royal decree, December 2003; implemented February 2004	5,370	Primary
Khoshnaw et al. (2024) [4]	Iraq (Koya)	Cross-sectional programme report	2020-2021	β-thalassaemia and sickle cell	Compulsory programme, Kurdistan Region, from 2008	2	Primary
Rouh AlDeen et al. (2021) [5]	Kuwait	Programme cohort	2009-2020	β-thalassaemia and sickle cell	Premarital Screening Law 31/2008, in force 2009	670	Primary
Bahram et al. (2023) [6]	Bahrain	Cross-sectional follow-up survey	NR	β-thalassaemia and sickle cell	National mandatory programme (instrument not stated; abstract-only)	159	Primary
Al-Allawi et al. (2013) [7]	Iraq (NE/Sulaimani)	Programme cohort	2008-2012	β-thalassaemia and sickle cell	Mandatory by law, Kurdistan Region, from 2008	107	Primary
Al-Allawi et al. (2015) [8]	Iraq (Duhok)	Programme cohort	2008-2012	β-thalassaemia and sickle cell	Regional parliament legislation, Kurdistan Region, 2008	198	Primary
Joulaei et al. (2014) [9]	Iran (Fars)	Cross-sectional programme report	1997-2011	β-thalassaemia	National mandatory premarital screening (programme from 1995)	4,563	Both
Khorasani et al. (2008) [10]	Iran (Mazandaran)	Programme cohort	1993-2005	β-thalassaemia	National law, March 1998 (as dated by the report)	3,329	Primary
Badedi et al. (2025) [11]	Saudi Arabia (Jazan)	Cross-sectional programme report	2021-2023	β-thalassaemia and sickle cell	Royal decree, December 2003; implemented February 2004	386	Primary

The most consequential shared feature was what the statute compelled. In most included reports, testing was compulsory while the subsequent marriage decision was not. Saudi Arabia issued marriage certificates irrespective of screening results [3]. In the Kurdistan programmes, reports sent to the court documented that testing had been performed without disclosing the result [7,8]. In Gaza, couples who declined to separate signed a declaration of personal responsibility before receiving a uniform marriage certificate without haematological information [2]. The Iranian programmes described the decision to marry or discontinue the marriage as remaining with the couple [9,10], while Khoshnaw et al. reported that couples signed to confirm that counselling had been provided and that some proceeded with marriage despite the result [4]. Kuwait was the only programme in which some couples faced a legal restriction on marriage, although this was contingent on the age of the female partner rather than solely on the genetic result [5]. Bozkurt reported compulsory testing but did not clearly describe whether the marriage decision remained unrestricted [1]. The legal position was not clearly stated in Bahrain and Jazan reports [6,11] (Tables 2-4).

**Table 2:** At-risk couples who did not proceed with marriage following statutory premarital screening Proportion of at-risk couples who did not proceed with marriage following identification of haemoglobinopathy carrier status through statutory premarital screening. Data are presented as the number of couples who did not proceed with marriage divided by the number of at-risk couples, with the corresponding percentage and exact 95% confidence interval. “Followed-up subset” indicates that the denominator comprised only couples for whom follow-up information regarding the marriage decision was available; “survey sample” indicates that the denominator comprised participants included in the survey; and “all identified” indicates that the denominator included all identified at-risk couples. Provenance indicates whether the data were obtained from the full text or an abstract. Risk of bias was assessed for the primary outcome. The naive total represents the crude sum across studies and should not be interpreted as a pooled estimate because study populations and ascertainment methods differed. The pooled estimate was calculated using a random-effects model.

Study	Country or region	Not proceeding/at-risk couples	%	95% CI	Denominator	Provenance	Risk of bias
Tarazi et al. (2007) [2]	Palestine (Gaza)	66/122	54.1	44.8-63.2	All identified	Full text	High risk
Memish and Saeedi (2011) [3]	Saudi Arabia	1,425/5,370	26.5	25.4-27.7	Follow-up subset	Full text	High risk
Khoshnaw et al. (2024) [4]	Iraq (Koya)	46,055	100	15.8-100.0	All identified	Full text	High risk
Rouh AlDeen et al. (2021) [5]	Kuwait	338/670	50.4	46.6-54.3	All identified	Full text	High risk
Bahram et al. (2023) [6]	Bahrain	52/159	32.7	25.5-40.6	Survey sample	Abstract	High risk
Al-Allawi et al. (2013) [7]	Iraq (NE/Sulaimani)	2/107	1.9	0.2-6.6	Follow-up subset	Full text	High risk
Al-Allawi et al. (2015) [8]	Iraq (Duhok)	19/198	9.6	5.9-14.6	Follow-up subset	Full text	High risk
Joulaei et al. (2014) [9]	Iran (Fars)	2,304/4,563	50.5	49.0-52.0	All identified	Full text	Moderate risk
Khorasani et al. (2008) [10]	Iran (Mazandaran)	1,826/3,329	54.9	53.1-56.6	All identified	Full text	Moderate risk
Badedi et al. (2025) [11]	Saudi Arabia (Jazan)	153/386	39.6	34.7-44.7	Survey sample	Full text	Moderate risk

**Table 3:** Incidence of affected births before versus after implementation of statutory premarital screening programmes Reported incidence of affected births before and after implementation of statutory premarital screening programmes. Data are presented as the reported number of affected births or newborns before and after programme implementation, the unit of measurement, the stated reduction, and the type of comparison. Before–after comparisons were not pooled because the studies used different outcome units, ascertainment methods, and comparison periods. Risk of bias was assessed using the ROBINS-I framework; risk-of-bias ratings are reported as stated in the review. NR, not reported; PND, prenatal diagnosis.

Study	Country	Comparison	Before → after	Unit	Stated reduction	Comparison type	PND	Termination	Risk of bias
Bozkurt (2007) [1]	Cyprus (North)	Pre-programme vs since 2002	18-20 → 0	Affected newborns per year (range as published)	1	Before-after	Yes	NR	Critical
Tarazi et al. (2007) [2]	Palestine (Gaza)	Pre-September 2000 vs 2005	15-20 → 4	Registered affected births per year	75%-80%	Before-after	No	No	Serious
Joulaei et al. (2014) [9]	Iran (Fars)	1997 vs 2011	101 → 2	New cases recorded per year	0.98	Before-after	Yes	Yes	Serious

**Table 4:** Programmes reporting both marriage decisions and affected births Comparison of the two included programmes that reported both the proportion of at-risk couples who did not proceed with marriage and the incidence of affected births before versus after programme implementation. The table also summarizes the availability of prenatal diagnosis and reported terminations of affected pregnancies. In the Fars province programme, prenatal diagnosis was available from 2000, and 800 of 806 affected fetuses detected were aborted. In Gaza, prenatal diagnosis was excluded from the programme by design because termination of pregnancy is prohibited in the community. The affected-birth data are presented using the units and time points reported in the respective studies.

Programme	At-risk couples not proceeding	Prenatal diagnosis	Terminations of affected pregnancies	Affected births
Gaza (Tarazi et al., 2007) [2]	66/122 (54.1%)	None — excluded from the programme by design	None	15-20/year (pre-2000) → 4/year (2005)
Iran, Fars province (Joulaei et al., 2014) [9]	2,304/4,563 (50.5%)	Available from 2000	800 of 806 affected fetuses detected were aborted	101/year (1997) → 2/year (2011)

Risk of Bias

Of the 10 studies contributing to the primary outcome, 7 were rated at high risk of bias and 3 at moderate risk; none were rated at low risk. The first major concern was incomplete coverage of the identified sample. Four studies reported the marital outcome only for couples they could trace, including the Saudi national programme, Bahrain, and both Kurdistan reports [3,6-8]. Five studies reported outcomes for every at-risk couple identified [2,4,5,9,10], while coverage could not be determined for the sampled cross-sectional Jazan study [11]. Joulaei et al. were the only authors to identify a non-ascertained group of couples who did not attend and retain them in the denominator [9]. The second major concern was sequential, sex-asymmetric screening. In one Kurdistan programme, the female partner was tested only when the male partner had abnormal red cell indices or a positive sickling test, meaning that some at-risk couples could remain unidentified [8]. Tarazi et al. used a similar approach in Gaza [2]. These screening algorithms therefore produce lower-bound estimates of the at-risk denominator, although the direction of their effect on the observed non-marriage proportion cannot be determined. All three studies contributing to the secondary outcome were rated at serious or critical risk on ROBINS-I, with confounding as the dominant domain (Tables 5, 6).

**Table 5:** Risk of bias for the primary outcome using the JBI checklist Risk of bias for the primary outcome (proportion of at-risk couples who did not proceed with marriage) was assessed using the JBI checklist for prevalence data. Individual domains were classified as yes, unclear, or no according to the checklist criteria. Overall risk of bias was classified as low, moderate, or high according to the prespecified assessment approach. Studies not included in the final systematic review reference list are presented without reference numbers. JBI, Joanna Briggs Institute.

Study	Sample frame	Sampling	Sample size	Description	Coverage of identified sample	Identification methods	Standard measurement	Analysis	Response rate	Overall
Memish and Saeedi (2011) [3]	Yes	Yes	Yes	Yes	No	Yes	Yes	Yes	No	High risk
Khoshnaw et al. (2024) [4]	Yes	Yes	No	Unclear	Yes	Yes	Yes	No	Yes	High risk
Rouh AlDeen et al. (2021) [5]	Yes	Yes	Yes	Yes	Yes	Yes	No	Yes	Yes	High risk
Bahram et al. (2023) [6]	Yes	Yes	Unclear	Unclear	No	Yes	Yes	Unclear	No	High risk
Al-Allawi et al. (2013) [7]	Yes	Yes	Yes	Yes	No	Yes	Yes	Yes	No	High risk
Al-Allawi et al. (2015) [8]	Yes	Yes	Yes	Yes	Unclear	No	No	Yes	Yes	High risk
Khorasani et al. (2008) [10]	Yes	Yes	Yes	Yes	Yes	Yes	Unclear	No	Yes	Moderate risk
Badedi et al. (2025) [11]	Yes	Unclear	Yes	Yes	Unclear	Yes	Yes	Yes	Unclear	Moderate risk

**Table 6:** Risk of bias for the secondary outcome using ROBINS-I Risk of bias for the secondary outcome (incidence of affected births before versus after implementation of the programme) was assessed using the ROBINS-I tool. D1, bias due to confounding; D2, bias in selection of participants into the study; D3, bias in classification of interventions; D4, bias due to deviations from intended interventions; D5, bias due to missing data; D6, bias in measurement of outcomes; D7, bias in selection of the reported result. Each domain was rated as low, moderate, serious, or critical risk of bias, with the overall rating determined according to the ROBINS-I framework. ROBINS-I, Risk of Bias in Non-randomised Studies of Interventions.

Study	D1: Confounding	D2: Selection	D3: Classification	D4: Deviations	D5: Missing data	D6: Outcome measurement	D7: Reported result	Overall
Bozkurt (2007) [1]	Serious	Moderate	Serious	Serious	Serious	Moderate	Serious	Critical
Tarazi et al. (2007) [2]	Serious	Moderate	Low	Moderate	Serious	Serious	Serious	Serious
Joulaei et al. (2014) [9]	Serious	Moderate	Serious	Serious	Moderate	Moderate	Moderate	Serious

Primary Outcome: At-Risk Couples Who Did Not Proceed With the Marriage

Ten study cohorts contributed to the primary outcome. Study-level proportions varied substantially, from very low levels in some cohorts to complete non-proceeding in the smallest cohort [4,7]. Among cohorts with larger denominators, the range remained wide.

The prediction interval was also extremely wide, indicating that the pooled proportion should not be interpreted as an estimate of what a typical premarital screening programme achieves. The three largest studies showed markedly different proportions of couples not proceeding with marriage, further supporting substantial between-programme heterogeneity [3,9,10].

All sensitivity and subgroup analyses were post hoc. These analyses produced similar pooled point estimates, but this apparent stability should not be interpreted as evidence of robustness given the substantial between-study heterogeneity. The prediction interval provides a more appropriate representation of the variability between programmes.

Secondary Outcome: Incidence of Affected Births

Three programmes reported affected births before and after programme implementation [1,9,2]. None were pooled because the underlying denominators, ascertainment methods, and comparison periods were not sufficiently comparable.

All three programmes reported substantial reductions in affected births following implementation of their screening strategies. However, each comparison had important limitations, including contaminated or undated baseline periods, differences in case ascertainment, and reliance on registered cases in some settings [1,9,2]. These limitations prevent attribution of the observed reductions solely to premarital screening.

The Two Pathways

Two programmes reported both marriage decisions and affected-birth outcomes, allowing limited examination of the potential pathways through which screening may operate.

In Fars province, Iran, approximately half of at-risk couples did not proceed with marriage. Prenatal diagnosis subsequently became available, and most affected fetuses identified through the programme were terminated. During the same period, recorded affected births declined substantially [9].

In Gaza, a similar proportion of at-risk couples did not proceed with marriage. Prenatal diagnosis was not incorporated into the programme because termination was prohibited, and no terminations occurred. Nevertheless, registered affected births declined substantially [2].

The Iranian findings are consistent with a shift from marriage avoidance towards reproductive intervention after prenatal diagnosis became available, but they do not establish a causal substitution between the two pathways. The Gaza findings indicate that reductions in affected births can occur even when prenatal diagnosis and termination are unavailable, although the evidence is insufficient to determine the mechanism.

Discussion

Principal Findings

This systematic review found very low-certainty evidence that statutory premarital haemoglobinopathy screening is associated with substantial variation in the proportion of at-risk couples who do not proceed with marriage. The findings were highly heterogeneous, indicating that a single pooled estimate is unlikely to represent a typical programme. Affected births decreased substantially in the programmes reporting before-and-after data, although these uncontrolled comparisons cannot establish causality. Overall, the findings suggest that programme effects depend strongly on the legal, reproductive, and cultural pathways available after an at-risk result.

Implications for Policy and Research

Premarital screening can influence affected births through two main pathways: couples may decide not to marry, or they may proceed with marriage and subsequently use prenatal diagnosis and termination. The included programmes appear to rely on different combinations of these pathways. Therefore, a programme introduced where prenatal diagnosis or lawful termination is unavailable should not automatically be considered ineffective; rather, its effect depends more heavily on counselling and subsequent marriage decisions. Programme evaluation should consequently include affected birth outcomes alongside marriage decisions because a declining non-marriage rate may reflect a shift towards reproductive options rather than programme failure. Future studies should report the complete identified at-risk denominator, distinguish legally prohibited marriage from voluntary decisions, and assess both partners rather than relying on sequential screening. Research should also directly compare programmes with and without accessible prenatal diagnosis and lawful termination.

Limitations

The evidence base and this review have substantial limitations. The search used major bibliographic databases but did not include regional databases, grey literature, trial registries, or citation searching, potentially under-representing regionally published programmes. Many potentially eligible reports were adjudicated from abstracts alone, and full-text assessment resulted in substantive corrections to several abstract-level extractions. All studies contributing to the outcomes were at moderate or high risk of bias, while the affected birth outcome was based on uncontrolled before-and-after comparisons. Interpretation of the mechanism is particularly limited because termination availability was incompletely reported and only one included programme clearly operated without prenatal diagnosis. Finally, overlap decisions may influence the pooled estimate, and reports from the same programme should not be interpreted as independent replications. These limitations warrant considerable caution in interpreting both the pooled estimate and differences between programmes.

## Conclusions

Statutory premarital haemoglobinopathy screening programmes may contribute to reductions in the number of affected children born, but the response of at-risk couples varies substantially, and no single pooled estimate of non-marriage is informative. In most programmes, the legal requirement is for screening rather than a particular marriage decision. The pathways available after an at-risk result, including reproductive options and counselling, appear to influence how couples respond. However, the certainty of the evidence is very low, and important limitations in the evidence base and review process restrict the strength and generalisability of these conclusions. The findings should therefore be regarded as a structured assessment of the current evidence rather than as a basis for programme design.
